# Do people who experience incarceration age more quickly? Exploratory analyses using retrospective cohort data on mortality from Ontario, Canada

**DOI:** 10.1371/journal.pone.0175837

**Published:** 2017-04-14

**Authors:** Fiona G. Kouyoumdjian, Evgeny M. Andreev, Rohan Borschmann, Stuart A. Kinner, Andres McConnon

**Affiliations:** 1 Department of Family Medicine, McMaster University, Hamilton, Ontario, Canada; 2 Centre for Urban Health Solutions, St. Michael’s Hospital, Toronto, Ontario, Canada; 3 Institute for Clinical Evaluative Sciences, Toronto, Ontario, Canada; 4 National Research University Higher School of Economics, Russian Federation; 5 Centre for Adolescent Health, Murdoch Childrens Research Institute (Royal Children’s Hospital), Melbourne, Australia; 6 Centre for Mental Health, Melbourne School of Population and Global Health, University of Melbourne, Melbourne, Australia; 7 Health Services and Population Research Department, Institute of Psychiatry, Psychology and Neuroscience, King’s College London, United Kingdom; 8 Griffith Criminology Institute, Griffith University, Brisbane, Australia; 9 School of Public Health and Preventive Medicine, Monash University, Melbourne, Australia; 10 Mater Research Institute, University of Queensland, St. Lucia, Australia; Leibniz Institute for Prvention Research and Epidemiology BIPS, GERMANY

## Abstract

**Objectives:**

We aimed to explore whether mortality data are consistent with the view that aging is accelerated for people with a history of incarceration compared to the general population, using data on mortality rates and life expectancy for persons in Ontario, Canada.

**Methods:**

We obtained data from the Ontario Ministry of Community Safety and Correctional Services on all adults admitted to provincial correctional facilities in Ontario in 2000, and linked these data with death records from provincial vital statistics between January 1, 2000 and December 31, 2012. We used life table methods to calculate mortality rates and life expectancies for this cohort by sex and 5-year age group. We similarly generated population comparison rates using publicly available data for the general population of Ontario in 2006 as the midpoint of the follow up period. We compared these mortality indices between the 2000 Ontario prison cohort and the general population by age group and sex.

**Results:**

The difference in all-cause mortality rates between the 2000 Ontario prison cohort and the general population was greatest for younger adults, with the prison cohort experiencing rates of death that would be expected for persons at least 15 years older at ages 20 to 44 for men and ages 20 to 59 for women. Life expectancy in the 2000 Ontario prison cohort was most similar to life expectancy of persons five years older in the general population at age intervals 20 to 45 in men and 20 to 30 in women.

**Conclusions:**

For most of adulthood, life expectancy and mortality rates are worse for adults with a history of incarceration than for the general population in Ontario, Canada. However, the association between mortality and incarceration status is modified by age, with the greatest relative burden of mortality experienced by younger persons with a history of incarceration and modified by sex, with worse relative mortality in women. Future research should explore the association between incarceration status and markers of aging including mortality, morbidity and physical appearance.

## Background

A commonly advanced assertion in research, clinical and policy settings is that people who experience incarceration age at a faster rate compared to the general population, and specifically that the physiological age of prisoners is 10 to 15 years greater than their chronological age [1–5]. The meaning of this claim is unclear, as accelerated aging could imply looking older [1] or having morbidity or mortality indices expected of older persons. Irrespective of the operational definition, there is a lack of evidence regarding whether people who experience incarceration age more quickly than those who do not experience incarceration.

The concept of physiological or biological age recognizes that individuals experience aging processes at different rates, and differences in aging may not be adequately represented by chronological age [6]. At the individual level, if a person had a higher level of an age-related parameter than would be expected for their chronological age peers, we would consider that person’s physiological or biological age to be higher than their chronological age. Extrapolating this concept to the population level, if members of one population had, on average, a higher prevalence or mean level of an age-related parameter than members of a comparator population, then we would consider the biological or physiological age of the first population to be increased relative to their chronological age.

Understanding whether, why and in what ways people who experience incarceration experience accelerated aging is important from health and public policy perspectives. Appearing older may affect life trajectories in diverse ways, for example affecting employment opportunities [7] or treatment by police, justice and health systems. Understanding the health status of this population is important for health care providers as they care for patients and for administrators to inform decision-making regarding health care services and institutional programming. Finally, there may be opportunities to intervene to prevent accelerated aging if it is occurring in this population.

To date there has been no empirical examination of the supposition that people who experience incarceration age more quickly compared to the general population. Our objective was to explore whether mortality data are consistent with the assertion that aging is accelerated for people who experience incarceration compared to the general population, using data on mortality rates and life expectancy. We conducted analyses using population-based data on people who had experienced incarceration and the general population in Ontario, Canada.

## Methods

### Population

We defined the “2000 Ontario prison cohort” as all persons who were admitted to a provincial correctional facility for adults in Ontario in 2000, regardless of the length of time spent in custody or whether they had been detained prior to sentencing (“remanded”) or sentenced (“incarcerated”); in this manuscript, we use the term “incarcerated” to include both groups. The 2000 Ontario prison cohort includes persons who were subsequently transferred to the federal system, *i*.*e*. those who were sentenced to two or more years in custody.

We defined the total population of Ontario, Canada as the general population.

### Data sources

The Ontario Ministry of Community Safety and Correctional Services (MCSCS) provided demographic data, health card numbers and information on deaths while under supervision for the 2000 Ontario prison cohort. These data were transferred from the MCSCS to the Institute for Clinical Evaluative Sciences (ICES), an independent, nonprofit organization funded by the Ontario Ministry of Health and Long-Term Care. We linked these persons to people in the Registered Persons Database, which is a roster of all people eligible for the Ontario Health Insurance Plan, and through the Registered Persons Database, we accessed a unique encrypted health card number that identifies individuals across databases at ICES, including the Ontario Registrar General Death database. We conducted deterministic linkage using health card numbers when provided by the MCSCS, or else probabilistic linkage using name, sex and date of birth, with clerical review of gray area matches when needed [8]. In the Ontario Registrar General Death database, we accessed data for the 2000 Ontario prison cohort on deaths in custody or in the community between January 1, 2000 and December 31, 2012. Details regarding data linkage and outcome definition are provided elsewhere [9].

For the general population, we accessed publicly available data from Statistics Canada for Ontario for 2006, which is the midpoint of the follow up period, including the number of deaths and persons at risk of death by age group and sex from the Canadian Socio-Economic Information Management System (CANSIM) Tables 102–0504 and 109–5325 [10], and life expectancy by age and sex from the Canadian Human Mortality Database [11].

### Analyses

We included data for all persons in the 2000 Ontario prison cohort, but excluded person-years of contribution to ages younger than 20 for men and women and to ages older than 79 for men and 69 for women because of limited data.

For the 2000 Ontario prison cohort, we used abridged life table methods to calculate mortality rates [12, 13], *i*.*e*. considering the number of deaths at each age and the number of years contributed to each age during the follow up period between 2000 and 2012. We calculated mortality rates and their 95% confidence limits by 5-year age group and by sex [14].

We compared mortality rates between the 2000 Ontario prison cohort and the general population. We did not calculate 95% confidence limits for the mortality rate point estimates for the general population, as we assumed that the sample size approached the population size, such that sampling error would be minimal and the confidence interval would approach zero [15]. We considered the difference between populations to be significant if the 95% confidence limit for the 2000 Ontario prison cohort did not overlap with the point estimate for the general population [16]. We identified the mortality rate in the general population that was most similar to—but not higher than (as a conservative measure)—the mortality rate in the 2000 Ontario prison cohort for each age group. We then calculated the difference in years of age between the age group for the 2000 Ontario prison cohort and the age group in the general population with the most similar mortality rate.

We calculated life expectancies by sex and at various age intervals for the 2000 Ontario prison cohort and the general population using abridged life tables [12, 13] and corresponding 95% confidence intervals [14]. The life expectancy refers to the number of years of life remaining at each age interval, and is derived based on the conditional probability of survival in each subsequent age interval. Similar to the comparison of mortality rates, we identified the age at which the life expectancy in the general population was most similar to—but not less than (as a conservative measure)—the life expectancy in the 2000 Ontario prison cohort for each age interval by sex.

The study was approved by the Ministry of Community Safety and Correctional Services Research Committee and by the St. Michael’s Hospital Research Ethics Board. Consistent with Article 5.5A of the Canadian Tri-Council Policy regarding the secondary use of data [17], no written or verbal consent was obtained from participants for the secondary use of these data.

## Results

We achieved linkage with health administrative data for 97.4% of persons in the 2000 Ontario prison cohort (48,166/49,470) [9]. The mean length of follow up was 12.0 years for men and 12.1 years for women in the 2000 Ontario prison cohort, and the majority of the follow up period was spent in the community and not in custody: 6.6% of person-years for men and 3.4% for women were spent in provincial custody and 4.8% of men and 3.3% of women were transferred to federal custody.

Table 1 shows the age distribution of persons in the 2000 Ontario prison cohort at the time of initial admission to provincial custody in 2000.

**Table 1:** Age distribution by sex of persons in the 2000 Ontario prison cohort* at the time of initial admission to provincial correctional facilities in Ontario in 2000.

Age group	Men N = 43,419 (%)	Women N = 4,747 (%)
15–19	4,054 (9.3)	411 (8.7)
20–24	8,257 (19.0)	752 (15.8)
25–29	6,349 (14.6)	684 (14.4)
30–34	6,942 (16.0)	878 (18.5)
35–39	6,936 (16.0)	901 (19.0)
40–44	5,009 (11.5)	567 (11.9)
45–49	2,805 (6.5)	306 (6.4)
50–54	1,565 (3.6)	117 (2.5)
55–59	771 (1.8)	74 (1.6)
60–64	395 (0.9)	32 (0.7)
65–69	206 (0.5)	21 (0.4)
70–74	85 (0.2)	≤5† (≤0.1)
75–79	37 (0.1)	≤5† (≤0.1)
≥80	8 (0.0)	≤5† (≤0.1)

*Persons admitted to provincial correctional facilities for adults in Ontario in 2000.

^†^To decrease the risk of identifying individuals, we indicated ≤5 as the number of people in cells in which there were 5 or fewer persons. For the percentage, we indicated the true percentage if the value didn’t change for numerators between 0 and 5, or else we used 5 as the numerator and indicated the value as less than or equal to the result.

**Fig 1:**
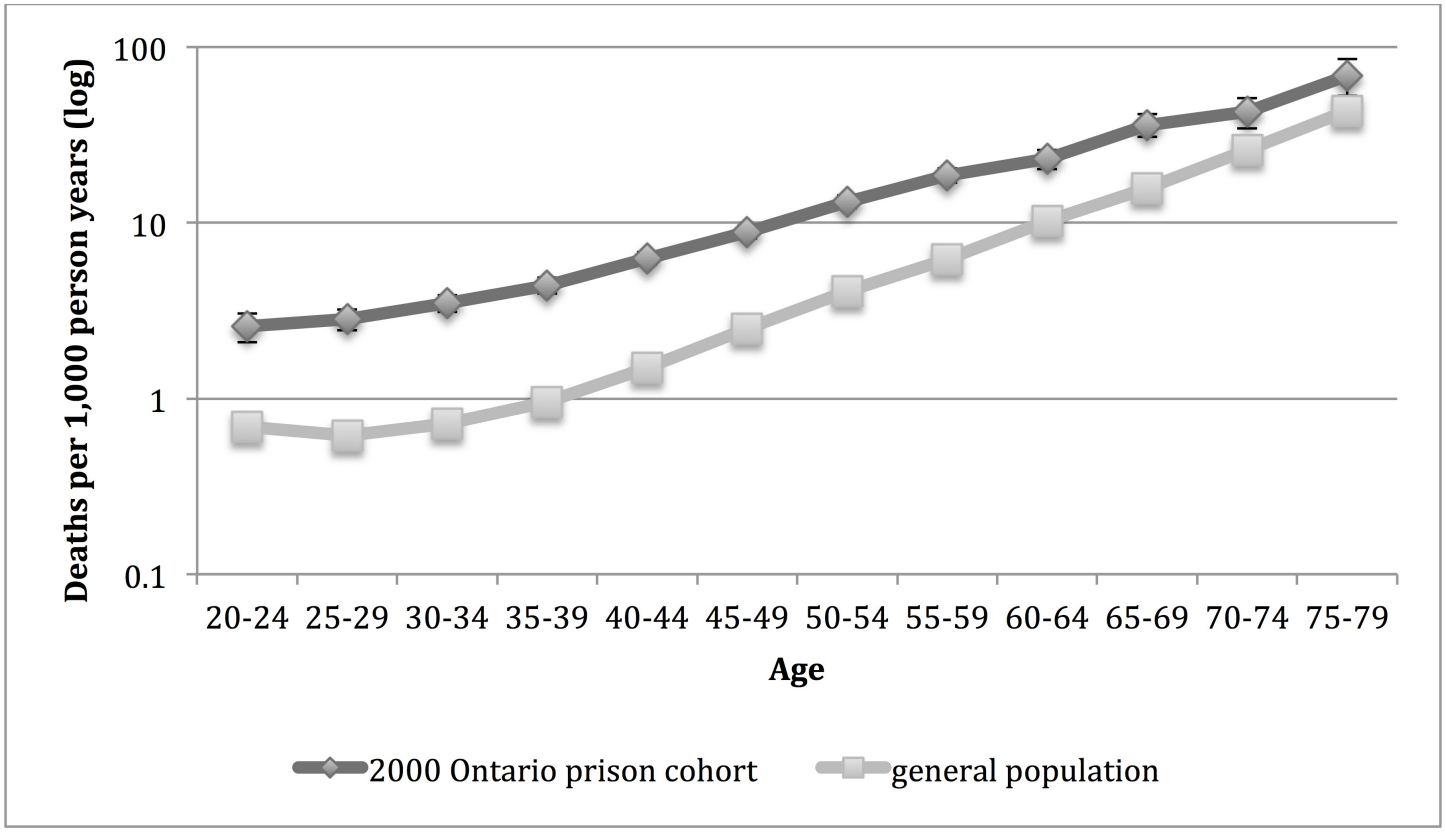
Mortality rate for men in the 2000 Ontario prison cohort* and general population† of Ontario by age group. *Men admitted to provincial correctional facilities for adults in Ontario in 2000 with follow up for death to 2012. 95% confidence intervals are indicated. †Death rate for men in Ontario population in 2006.

The mortality rate for men was significantly higher in the 2000 Ontario prison cohort compared to the general population for all age groups (Fig 1).

**Fig 2:**
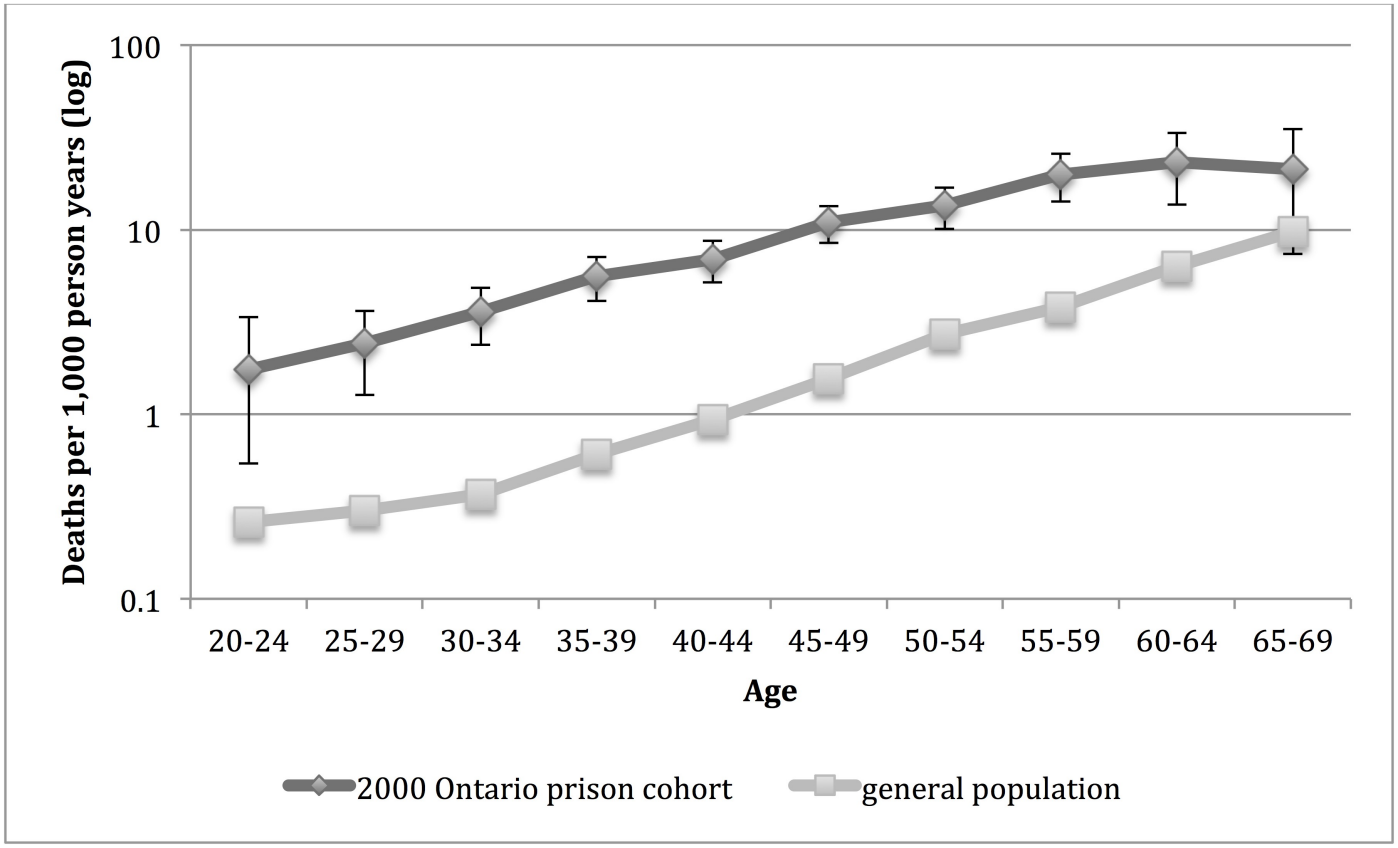
Mortality rate for women in the 2000 Ontario prison cohort* and general population† of Ontario, by age group. *Women admitted to provincial correctional facilities for adults in Ontario in 2000 with follow up for death to 2012. 95% confidence intervals are indicated. †Death rate for women in Ontario population in 2006.

For women, the mortality rate was significantly higher in the 2000 Ontario prison cohort than in the general population for age groups 20–24 to 60–64 and there was no significant difference between the cohort and general population for those in age group 65–69 (Fig 2).

For each sex, we identified the general population age group with the death rate that was closest to (but not higher than) the death rate for each age group in the 2000 Ontario prison cohort and then calculated the difference in years between these two age groups (Table 2). For men in the 2000 Ontario prison cohort, the death rate was closest to the general population death rate of persons 25 years older for those in age group 20–24; 20 years older for those in age group 25–29; 15 years older for those aged 30–34, 35–39 and 40–44; 10 years older for those aged 45–49, 50–54 and 55–59; 5 years older for aged 60–64 and 65–69; and the same age for those aged 70–74 and 75–79. For women in the 2000 Ontario prison cohort, the death rate was closest to the general population death rate for persons 25 years older for those aged 20–24; 20 years older for those between ages 25 and 49; 15 years older for those aged 50–54 and 55–59; 10 years older for those aged 60–64; and 5 years older for those aged 65–69.

**Table 2:** Comparing mortality rates across age groups between the 2000 Ontario prison cohort* and general population† of Ontario, by sex.

age group	Men			Women		
	death rate per 1,000 person years (95% CI)		Δ years for death rate‡	death rate per 1,000 person years (95% CI)		Δ years for death rate‡
	2000 Ontario prison cohort	general population		2000 Ontario prison cohort	general population	
20–24	2.6 (2.1, 3.0)	0.7	25	1.7 (0.5, 3.4)	0.3	25
25–29	2.8 (2.4, 3.2)	0.6	20	2.4 (1.3, 3.6)	0.3	20
30–34	3.5 (3.1, 3.9)	0.7	15	3.6 (2.4, 4.8)	0.4	20
35–39	4.4 (3.9, 4.9)	1.0	15	5.6 (4.1, 7.1)	0.6	20
40–44	6.3 (5.8, 6.8)	1.5	15	6.9 (5.2, 8.7)	0.9	20
45–49	8.9 (8.1, 9.6)	2.5	10	11.0 (8.5, 13.4)	1.6	20
50–54	13.2 (12.1, 14.3)	4.1	10	13.5 (10.1, 16.9)	2.7	15
55–59	18.6 (16.9, 20.4)	6.2	10	19.9 (14.2, 25.9)	3.8	15
60–64	23.1 (20.2, 26.0)	10.3	5	23.3 (13.7, 33.4)	6.4	10
65–69	36.1 (30.9, 41.5)	15.8	5	21.3 (7.4, 35.1)	9.7	5§
70–74	42.9 (34.5, 51.2)	26.1	0	-	16.3	-
75–79	69.1 (52.8, 85.6)	43.3	0	-	60.9	-

*Persons admitted to provincial correctional facilities for adults in Ontario in 2000 with follow up for death to 2012.

^†^Death rate for Ontario population in 2006.

^‡^The difference in years between the age group for the 2000 Ontario prison cohort and the corresponding age group for the general population that has the death rate that is most similar but not higher.

^§^The difference in mortality rates between the 2000 Ontario prison cohort and general population for this age group was not statistically significant.

**Fig 3:**
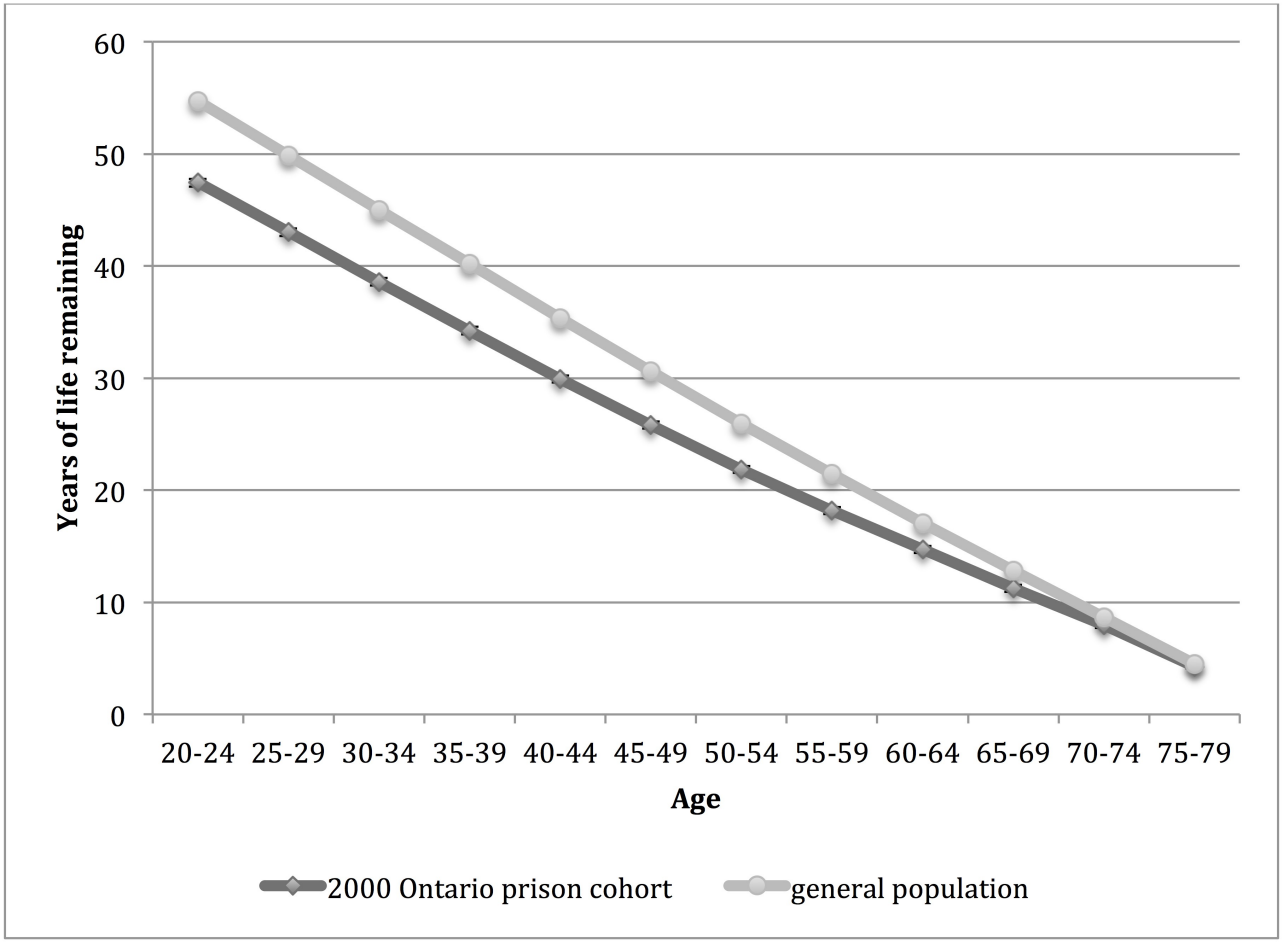
Life expectancy for men in the 2000 Ontario prison cohort* and general population† of Ontario, by age interval. *Men admitted to provincial correctional facilities for adults in Ontario in 2000 with follow up for death to 2012. 95% confidence intervals are indicated. †Life expectancy for men in the Ontario population in 2006.

**Fig 4:**
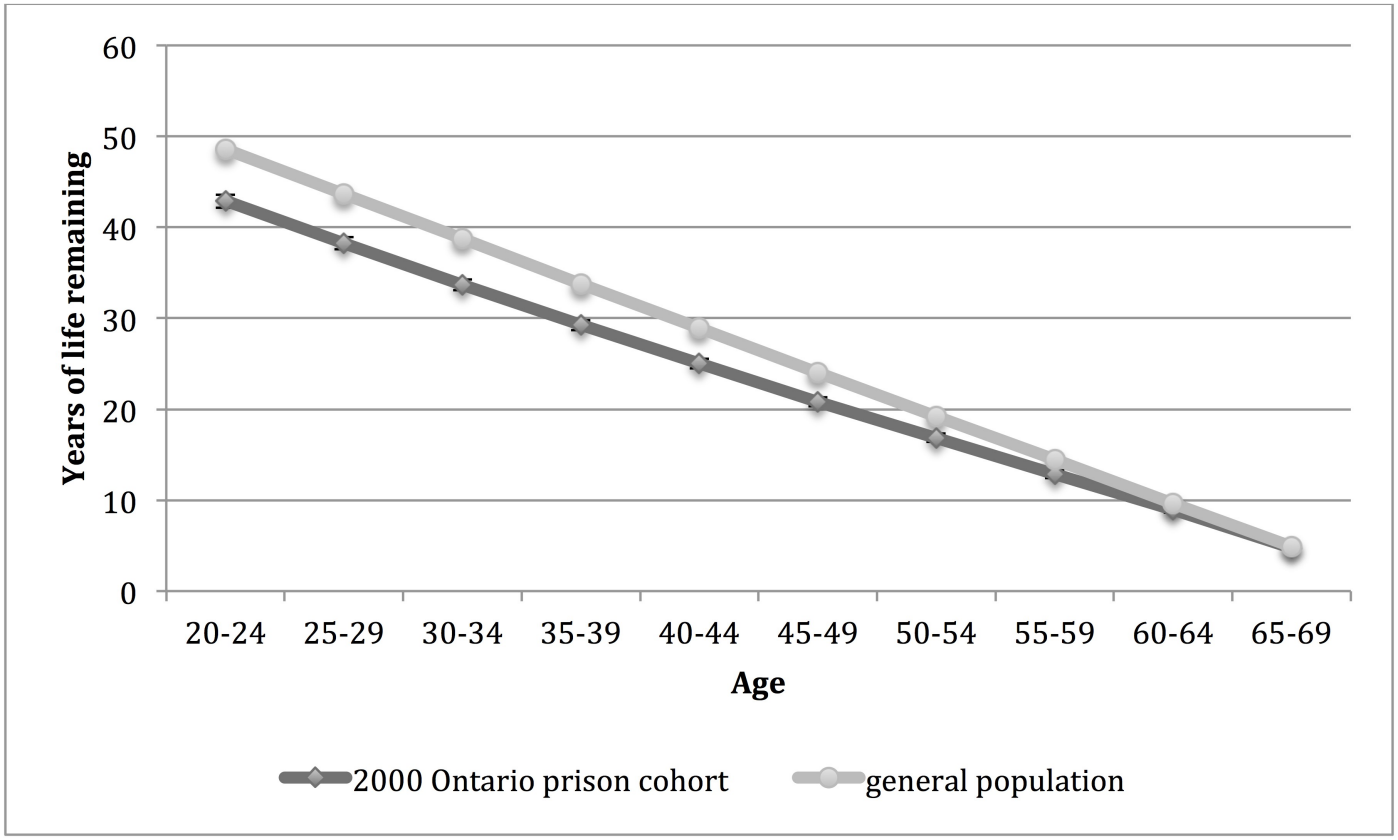
Life expectancy for women in the 2000 Ontario prison cohort* and general population† of Ontario, by age interval. *Women admitted to provincial correctional facilities for adults in Ontario in 2000 with follow up for death to 2012. 95% confidence intervals are indicated. †Life expectancy for women in the Ontario population in 2006.

Life expectancies for men and women in the 2000 Ontario prison cohort and general population are shown in Figs 3 and 4, respectively. Life expectancy was significantly lower for men in the 2000 Ontario prison cohort compared to the general population at age intervals 20 to 79 and for women at age intervals 20 to 60. There was no significant difference in life expectancy between groups for women at age interval 65. Comparing those in the 2000 Ontario prison cohort and the general population, the difference in life expectancy at age 20 was 7.3 years for men and 5.7 years for women.

The life expectancy of persons in the 2000 Ontario prison cohort was most similar to (but not lower than) the life expectancy for person in the general population five years older at age intervals 20 to 45 for men and for women at age intervals 20 to 30. The life expectancy was most similar to (but not lower than) the life expectancy in the general population for those at the same age interval for men at age intervals 50 to 79 and for women at age intervals 35 to 69.

## Discussion

As indicated by mortality rates and life expectancy, mortality risk is greater for persons who experience incarceration than for the general population across most of adulthood. However, the increased mortality burden is not evenly distributed across age groups or by sex. Younger adults and women who experience incarceration in Ontario have a particularly high burden of mortality relative to the general population. For persons aged 20 to 24, men and women in the 2000 Ontario prison cohort had mortality rates that would be expected for persons in the general population who were 25 years older. Further, men aged 25 to 44 and women aged 25 to 59 in the 2000 Ontario prison cohort had mortality rates that would be expected for persons in the general population who were 15 to 20 years older. Similarly, for both men and women, the difference in life expectancy between persons in the 2000 Ontario prison cohort and the general population was greatest at younger ages.

These findings are consistent with the view that across most of adulthood, persons who experience incarceration experience accelerated aging, as indicated by mortality data. However, adjusting a prisoner’s age uniformly by 10 to 15 years (as the commonly advanced assertion suggests) would be overly simplistic, as age and sex seem to modify the effect of a history of incarceration on mortality rate and life expectancy: a history of incarceration was associated with the greatest relative mortality burden in early adulthood and women with a history of incarceration had particularly high mortality rates [18].

There are several limitations to this study that may affect internal and external validity. Regarding internal validity, the follow up period for this retrospective cohort was long at over 12 years, which is important because mortality rates are higher in the immediate post-release period [9, 19–21] and overrepresentation of high risk periods would lead to underestimation of life expectancy for the 2000 Ontario prison cohort. However, the estimates of life expectancy for the 2000 Ontario prison cohort assume that the risk of death for someone at age 70 during the follow up period is the same as the risk of death would be at age 70 for someone currently in their 20s, *i*.*e*. assumes the absence of period and cohort effects [12], which is likely inaccurate and may lead to an underestimation of life expectancy. Studies with even longer follow up periods would more accurately estimate long-term mortality rates and associated life expectancy of prisoners. As there were relatively few person-years in the youngest and oldest age groups, we limited our analyses to men aged 20 to 79 and women aged 20 to 69, recognizing that very small sample sizes may yield unstable estimates. We note that the sample sizes for the older age groups that we included may not have adequate power for direct comparison between the 2000 Ontario prison cohort and the general population, especially for women. We are unable to assess the validity of the probabilistic matching for this population with available data. Regarding external validity, the relationship between age and mortality indices in people who have experienced incarceration may vary by region and country. Further, these data on persons who were incarcerated in 2000 may no longer accurately reflect the mortality rates of persons currently incarcerated.

At a conceptual level, the use of all-cause mortality as an indicator of aging is imperfect, and the significance of this limitation depends on how one defines aging. If we define aging as the “persistent decline in the age-specific fitness components of an organism due to internal physiological deterioration” [22], then all-cause mortality may not be an appropriate indicator of aging since a large proportion of deaths in this population is due to external causes such as overdose and suicide as opposed to a gradual process contributing to physiological deterioration [9]. However, if we define aging as “a progressive increase throughout life, or after a given stadium, in the likelihood that a given individual will die, during the next succeeding unit of time, from randomly distributed causes” [23], then all-cause mortality would be an appropriate indicator of aging.

With these limitations in mind, further research is indicated to elucidate the association between a history of incarceration and aging. First, researchers should reproduce these analyses in other jurisdictions using mortality data with long-term follow up. Subject to sample size considerations, this should include analyses of death from so-called “natural” [24] causes such as cancer and cardiovascular diseases and exclude deaths from intentional and unintentional injuries, as well as all-cause mortality. Second, studies should be conducted to assess whether morbidity indices such as prevalence suggest accelerated aging in people who experience incarceration, including for chronic diseases. Third, researchers should conduct studies to examine whether prisoners appear older than their chronological age from the perspectives of relevant persons such as police and correctional officers, health care workers, and potential employers.

In addition to suggesting a need for further research on whether people who experience incarceration age prematurely, our study adds to a growing body of literature documenting increased burden of mortality in people who experience incarceration. There is an urgent need for tailored programs, policies and services in custody and post-release to reduce mortality in this population.

We issue two important cautions regarding the interpretation of these results. First, while the ecologic fallacy is implied in this discussion [12], even if people with a history of incarceration have a mean life expectancy which is significantly lower than the life expectancy for the general population, this does not mean that an individual prisoner or person with a history of incarceration will die at an earlier age than an individual without such a history. Clearly, individual behavioural, social and genetic factors contribute to mortality, morbidity and physical appearance. The erroneous interpretation of aggregate data regarding prisoner status and life expectancy could lead to harm through precipitating behaviours of individuals with a history of incarceration or contributing to inappropriate assessment and management of patients by health care providers. Second, this study did not assess the specific contribution of incarceration to mortality or aging in this population. Instead, we explored whether people who experience incarceration experience accelerated aging, which could be attributable to factors antecedent to incarceration, during incarceration, or subsequent to incarceration. For example, there is overrepresentation in the prison population of persons with low income status, persons with low educational attainment and Indigenous persons [25], each of which is associated with higher mortality rates and decreased life expectancy [10]. Of note, we followed persons in the 2000 Ontario prison cohort for up to 12 years after the index admission to custody, and the vast majority of the follow up time was spent in the community and not in custody.

In conclusion, these exploratory analyses suggest that people with a history of incarceration do experience accelerated aging; however, the common assertion that their physiological age is 10 to 15 years greater than their chronological age is not accurate for all age groups or for both sexes. In an age of evidence-informed health care and public policy, we recommend further work to define the association between incarceration status and aging, which could inform appropriate health care, services and interventions for people who experience incarceration, both in custody and after they return to the community.
